# Reminiscent music therapy combined with robot-assisted rehabilitation for elderly stroke patients: a pilot study

**DOI:** 10.1186/s12984-024-01315-y

**Published:** 2024-01-30

**Authors:** Qian Liu, Li Liu, Zuoyan Liu, Yang Xu, Fang Wang, Hong Cheng, Xiuying Hu

**Affiliations:** 1 Innovation Center of Nursing Research, Nursing Key Laboratory of Sichuan Province, West China Hospital, West China School of Nursing, Sichuan University, Sichuan University, Chengdu, China; 2 Department of Rehabilitation Medicine Center, West China Hospital, School of Nursing, Sichuan University/West, Sichuan University, Chengdu, China; 3 Department of Rehabilitation Medicine Center, West China Hospital, Sichuan University, Chengdu, China; 4 School of Automation Engineering, University of Electronic Science and Technology of China, Chengdu, China

**Keywords:** Stroke, Music therapy, Reminiscence therapy, Robotics, Rehabilitation

## Abstract

**Background:**

Although some studies suggest that robot-assisted technology can significantly improve upper limb function in stroke patients compared to traditional rehabilitation training, it is still necessary to incorporate an auxiliary intervention to alleviate negative emotions, thereby alleviating the post-stroke fatigue and encouraging patients to actively respond to rehabilitation. However, the effect of the auxiliary intervention is unknown.

**Objective:**

To evaluate the effect of reminiscent music therapy combined with robot-assisted rehabilitation in elderly patients with upper limb dysfunction.

**Methods:**

From November 2022 to March 2023, elderly patients with upper limb dysfunction after stroke were assigned to one of three groups, with group A receiving usual rehabilitation treatment and care plus robot-assisted rehabilitation and reminiscent music therapy, group B receiving usual rehabilitation treatment and care plus robot-assisted rehabilitation, and group C receiving only usual rehabilitation treatment and care. Thirty patients completed this study, with 10 participants in each group. Activities of daily living, self-esteem, rehabilitation self-efficacy, positive emotion and upper limb function were measured before and after the intervention. One-way analysis of variance, paired-sample t-test, Kruskal-Wallis H test, Wilcoxon signed rank sum test and Chi-square test were used to analyze the data.

**Results:**

According to the intragroup comparisons, in the three groups, all outcome measurements were significantly higher than those at baseline (all *P* < 0.05). After the intervention, the differences in the self-management effectiveness, rehabilitation self-efficacy, and positive emotion score were statistically significant among the three groups (all *P* < 0.05). In accordance with the results of Bonferroni analysis, the self-management effectiveness score of group A was significantly higher than that of Group B and Group C (all *P* < 0.05). The rehabilitation self-efficacy score of group A was significantly higher than that of Group B and Group C (*P* < 0.05). The positive emotion score of group A was significantly higher than that of Group B and Group C (*P* < 0.05).

**Conclusion:**

Reminiscent music therapy combined with robot-assisted rehabilitation is a promising approach to improve rehabilitation self-efficacy and positive emotion, which is evidence that reminiscent music therapy may be an effective auxiliary intervention to improve rehabilitation outcomes.

## Introduction

Stroke is one of the main causes of disability in the current world [1], which is the main cause of the burden of neurological disease in elderly people [2]. After stroke, there are varying degrees of functional impairment, with 80% of patients exhibiting upper limb motor dysfunction [3]. However, less than 20% of patients can partially recover upper limb motor function within 6 months of onset [4]. Upper limb dysfunction after stroke seriously affects patients’ daily life, learning and work, and greatly reduces their quality of life [5]. Therefore, timely and effective upper limb rehabilitation training is of great significance to improve the quality of life of stroke patients and reduce the economic burden on families and society. In recent years, robot-assisted technology has become a research hotspot in the rehabilitation of upper limb function in stroke patients [6]. Robot-assisted training contains the key elements of motor learning, which can provide interactive, quantifiable and highly repetitive training programs for hemiplegic patients [7]. Compared with traditional rehabilitation training, upper limb rehabilitation robot can provide stroke patients with high-intensity, high-repeatability and accurate active rehabilitation training to reshape neural function and motor function, to help the recovery of brain injury, and to promote the reorganization of the motor cortex of the brain [8–10]. However, in addition to differences in rehabilitation modalities, there are other factors that affect rehabilitation outcomes. Patients are prone to exhibiting negative emotions and negative coping styles after stroke [11], which may easily lead to the emergence of post-stroke fatigue, with an incidence rate of 25–85% [12, 13]. Post-stroke fatigue has a negative impact on patients’ quality of life and independence [14], affecting the subjective perception of autonomous activities and the susceptibility to exercise fatigue [15], leading to poor rehabilitation prognosis [14]. For this reason, it is necessary to incorporate auxiliary interventions in the upper limb rehabilitation process to alleviate the negative emotions of stroke patients, such as depression and anxiety, thereby alleviating the level of post-stroke fatigue, encouraging patients to actively respond to functional rehabilitation treatment, and ultimately improving the effects of rehabilitation.

Music have a positive impact on hypokinesia and music therapy can facilitate and modulate neural plasticity, and easily elicit the interactions between perception and action systems, which is one of the key elements of neurological rehabilitation [16, 17]. In addition, previous studies have shown that music can induce activation of the motor regions together with the auditory cortex, indicating that music is able to induce the process of brain reorganization and enhance the neuronal co-activation and functional coupling of the auditory-motor network after stroke [16, 18]. Moreover, music is helpful to reorganize and integrate information processing, executive control, and emotion, thereby shifting attention away from fatigue and promoting the acquisition of motor skills [19, 20]. Music therapy is beneficial for several motor-cognitive functions, which may be an appropriate method for neurological rehabilitation after stroke [21, 22]. Reminiscent music can help elderly patients summon autobiographical memory and recall, which are central to improve the level of self-esteem, mastery, and self-efficacy [23]. Music is one of the most common media for evoking nostalgia [24], and reminiscence intervention has been proven to be beneficial to improve positive attitude and hope level of elderly stroke patients [25, 26]. It seems that music may play two roles in summoning memories, meaning that it promotes the retrieval of autobiographical memories and enhances the vividness, accessibility, and impact of the retrieved memories [27]. Some scholars investigated the neurochemistry of music and found that music therapy improved markers of innate immunity, and it is plausible that these benefits may be observed when music and nostalgia are combined and used for the elderly [27, 28].

Population ageing has become a global public health problem [29], and the World Health Organization proposed that the healthy life expectancy would be more significant than the average life expectancy [30]. Meanwhile, independent physical and cognitive abilities are considered key components of healthy life expectancy for elderly to achieve successful ageing [30]. Therefore, we combined reminiscent music therapy with robot-assisted rehabilitation, and preliminarily explored the impact of this intervention on elderly patients after stroke through clinical trials, to provide a new idea for the clinical exploration of a safe and effective non-pharmacological therapy.

## Methods

### Settings and participants

This single-blind, three-arm randomized controlled pilot trial was conducted from November 2022 to March 2023 in the Department of Rehabilitation Medicine Center of West China Hospital of Sichuan University. This trial was approved by the Ethics Committee of West China Hospital of Sichuan University (number: 2022 − 852) and was registered on the Chinese Clinical Trial Registry (ChiCTR2200063738).

Elderly patients with upper limb dysfunction after stroke participated in this pilot study. The inclusion criteria were: diagnosed with stroke; 60 years old and above; with upper limb dysfunction; conscious and in stable condition with stable vital signs; normal cognitive function with score of mini-mental state examination ≥ 27; normal attention, vision, hearing and communication judged by communication; no music-related learning experience and accept music; participate in this study voluntarily and sign the informed consent form. Exclusion criteria were: with severe primary diseases of the heart, liver, kidney and hematopoietic system; with a history of mental disease; joint dislocation or skin damage of the upper limb; excessive area of cerebral infarction or cerebral hemorrhage; and uncooperative participants.

### Sample size

A general flat rule of a pilot study is to recruit at least 30 participants to estimate a parameter [31–33]. In addition, some scholars have suggested that the minimum sample size is 12 participants per treatment group [34]. and previous works have suggested that the sample size of this study is feasible for a pilot study. Therefore, we finial decided that the sample size was 30 based on these recommendations, which was suggested by previous works to be feasible for a pilot study.

### Randomization and blinding

Participants were randomly assigned to receive usual rehabilitation treatment and care plus robot-assisted rehabilitation and reminiscent music therapy (group A), usual rehabilitation treatment and care plus robot-assisted rehabilitation (group B), or usual rehabilitation treatment and care (group C). For randomization, the random number table was generated in advance by SPSS 22.0 software. To ensure the full concealment of allocation, randomized allocation was carried out by a person who was not involved in the intervention. The data collector and study statistician were blinded to the participants’ allocation.

### Intervention

#### Usual rehabilitation treatment and care

All patients received usual rehabilitation treatment and care for stroke every day during hospitalization. Usual rehabilitation treatment mainly included drug treatment, comprehensive training of hemiplegic limbs, physical therapy, acupuncture and hyperbaric oxygen therapy. Usual rehabilitation care included basic environmental care, diet care, drug care, health education, psychological care, and rehabilitation care. There was no extra conventional therapy time to make up for the time spent on robotic therapy.

#### Robot-assisted rehabilitation

In accordance with the specific conditions of the patients, the rehabilitation therapists developed an appropriate scheme to carry out upper limb rehabilitation training lasting for 40 min once a day, five times per week for 3 weeks. The robot equipment used in this study was produced by Guangzhou Yikang medical equipment industry Co Ltd. It is a three-dimensional upper limb rehabilitation robot (model: A6), with five training modes: passive mode, active-passive mode, active mode, prescription mode and track editing mode. The specific rehabilitation training tasks were achieved through playing games. Rehabilitation therapists assessed the level of limb function of stroke patients and matched them with appropriate training modes and games, such as WEATHER GIRL, CUT FRUITS, AVOID OBSTACLES, EAT COINS, Whac-A-Mole, and so on. Each game can be played in the five training modes. During the intervention period, appropriate game changes were made for stroke patients to maintain their interest in training.

#### Reminiscent music therapy

This study used music to stimulate patients to develop a sense of nostalgia. One hundred Chinese songs released from 1935 to 1980 were selected through preliminary surveys to ensure that elderly aged 60 and above were familiar with these songs when they were young. Songs were divided into 10 song lists and each list had 10 reminiscent songs. Each time the robot-assisted rehabilitation training of upper limb was carried out, a reminiscent song list was played for patients with a wireless headset. Music was heard contemporaneously with the robotic therapy. After the training, the patients were asked to sit down and concentrate on breathing for 2 min.

### Assessment

Demographic data, such as gender, age, educational level, marital status, residence, nationality, religion, monthly income per capita of family, type of medical insurance, diagnosis, type of combined chronic disease (hypertension, diabetes, and coronary heart disease), Brunnstrom stage and main caregivers, were collected before intervention. A research assistant who did not know the randomization scheme collected the outcomes before (T0) and after intervention (T1). The primary outcome was the activities of daily living, and secondary outcomes included self-esteem, rehabilitation self-efficacy, positive emotion and upper limb function.

The modified Barthel Index with 10 items was used to measure the activities of daily living [35]. Each item is divided into five levels according to the degree of dependence. The total score ranges from 0 to 100. The higher the total score, the better the self-care ability.

Self-esteem was evaluated by the Self-Esteem Scale compiled by Rosenberg in 1965 [36]. This scale has 10 items and each item of the Chinese version of Self-Esteem Scale is scored from 1 (quite wrong) to 4 (quite right). The total score ranges from 10 ~ 40, and a higher total score indicates a higher sense of self-esteem.

The Stroke Self-Efficacy Questionnaire compiled by Jones et al. in 2008 was used to examine the level of rehabilitation self-efficacy [37]. Li et al. translated and revised it into Chinese with 2 dimensions including daily life activity effectiveness and self-management effectiveness, and 11 items [38]. Each item is scored from 1 (very unconfident) to 10 (very confident). The total score ranges from 11 to 110, and a higher total score indicates a higher sense of rehabilitation self-efficacy.

The Positive Affect and Negative Affect Scale developed by Watson et al. in 1988 and revised by Qiu et al. in 2008 was used to measure the level of positive emotion [39]. We only used the positive emotion dimension with 9 items. Each item is scored from 1 (never) to 5 (almost). The total score ranges from 9 to 45, and a higher total score indicates more positive emotions.

Upper limb function was measured by the Fugl-Meyer Assessment with the advantages of reliability and high sensitivity [40]. The upper limb dimension of the simplified Fugl-Meyer Assessment has 33 items. Each item is scored from 0 (cannot complete) to 2 (complete). The total score ranges from 0 to 66. The higher the total score, the better the motor function of the upper limbs and hands.

### Statistical analysis

IBM SPSS Statistics 22.0 was used to analyze the data. A P-P diagram was used to test the normal distribution of measurement data. Normally distributed measurement data were expressed as the mean ± standard deviation (SD); otherwise, the median and quartile were used. When measurement data followed a normal distribution and homogeneity of variance, one-way analysis of variance (ANOVA) was used for intergroup comparisons and a paired-sample t-test was used for intragroup comparisons. Otherwise, the Kruskal-Wallis H test was used for intergroup comparisons, and the Wilcoxon signed rank sum test was used for intragroup comparisons. If the results of ANOVA had significant differences, the Bonferroni post hoc test was used for multiple comparisons. Counting data were described by number and percentage. Chi-square test and Fisher’s exact test were used to analyze the counting data. Statistical significance was defined as *P* < 0.05.

## Results

### Baseline characteristics of participants

**Fig. 1:**
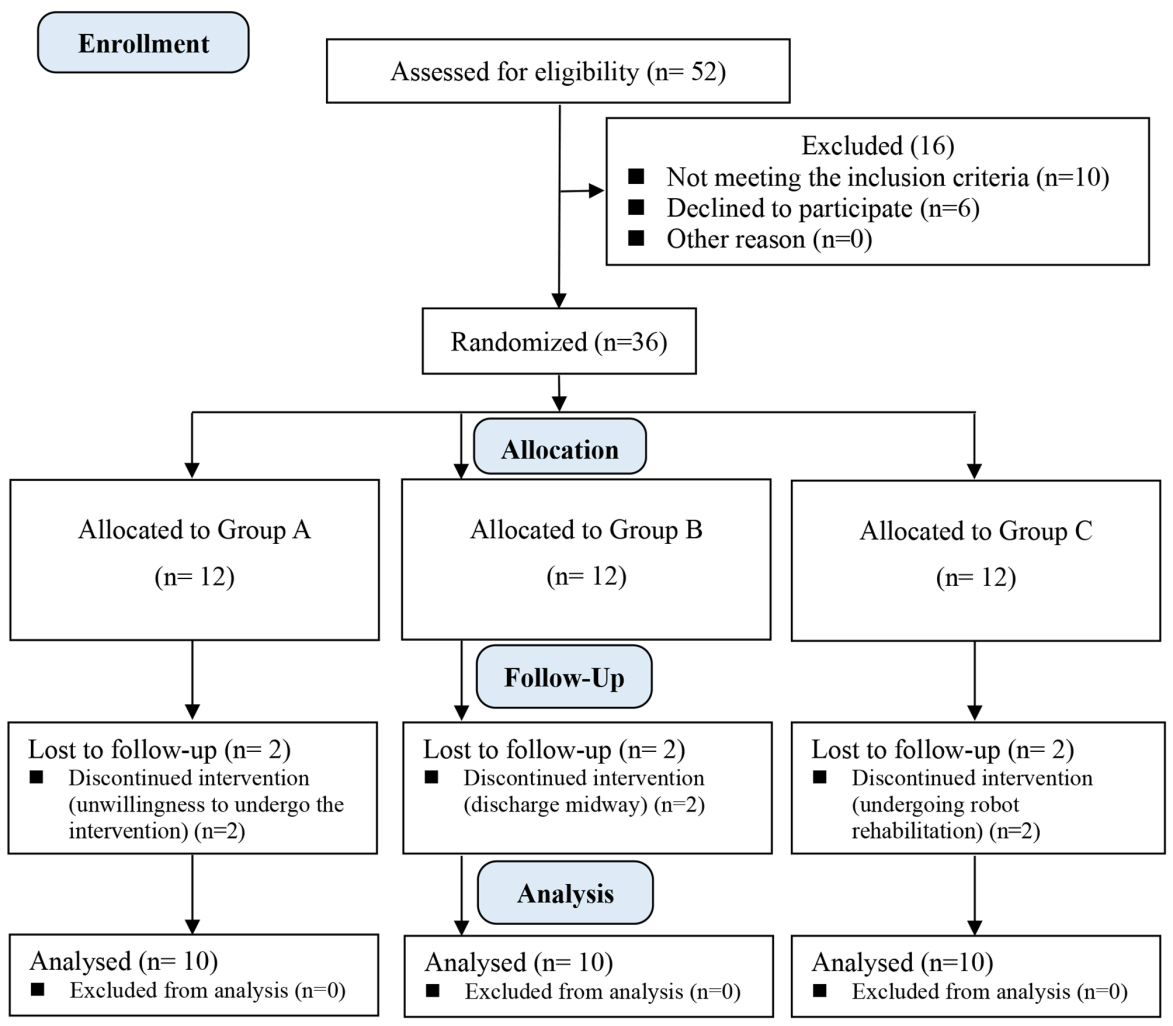
The consolidated standards of reporting trials flow diagram of this study

As shown in Fig. 1, of the 52 eligible patients, 10 did not meet the inclusion criteria and 6 refused to take part in the study. Finally, a total of 36 participants were recruited and each group consisted of 12 patients. During the intervention process, two participants were excluded from group C due to undergoing robot rehabilitation, two participants from group A were unwilling to undergo the robotic therapy because they did not want to bear the cost of robotic therapy, and two participants were excluded from group B due to discharge midway. Finally, 30 participants were included in this pilot study and 10 patients were included in each group, including 21 men and 9 women. Table 1 shows the participants’ demographics, and there was no significant difference in the baseline data between the three groups (*P* > 0.05).

**Table 1:** Baseline demographics of 30 participants

Items	Group A (*n* = 10)	Group B (*n* = 10)	Group C (*n* = 10)	F/χ^2^	*P*
Age ^a^; mean ± SD	67.60 ± 8.96	69.10 ± 9.46	65.60 ± 8.09	0.453	0.640
Gender ^b^; n (%)				0.000	1.000
Male	7 (70.0)	7 (70.0)	7 (70.0)		
Female	3 (30.0)	3 (30.0)	3 (30.0)		
Education ^b^; n (%)				2.865	0.581
Junior high school and below	2 (20.0)	3 (30.0)	3 (30.0)		
Senior high school	3 (30.0)	5 (50.0)	5 (50.0)		
College and above	5 (50.0)	2 (20.0)	2 (20.0)		
Marital status ^b^; n (%)				1.920	0.383
Widowed	1 (10.0)	3 (30.0)	1 (10.0)		
Married	9 (90.0)	7 (70.0)	9 (90.0)		
Residence ^b^; n (%)				1.875	0.392
Rural	3 (30.0)	6 (60.0)	5 (50.0)		
Urban	7 (70.0)	4 (40.0)	5 (50.0)		
Monthly income per capita (RMB) ^b^; n (%)				5.143	0.273
< 3000	1 (10.0)	3 (30.0)	4 (40.0)		
3000–5000	4 (40.0)	5 (50.0)	5 (50.0)		
≥ 5000	5 (50.0)	2 (20.0)	1 (10.0)		
Medical insurance ^b^; n (%)				0.800	0.670
New Rural Cooperative medical insurance	4 (40.0)	6 (60.0)	5 (50.0)		
Urban resident medical insurance	6 (60.0)	4 (40.0)	5 (50.0)		
Diagnosis ^b^; n (%)				3.732	0.155
Ischemic stroke	7 (70.0)	8 (80.0)	4 (40.0)		
Hemorrhagic stroke	3 (30.0)	2 (20.0)	6 (60.0)		
Hemiplegia ^b^; n (%)				2.500	0.287
Left upper limb	6 (60.0)	3 (30.0)	3 (30.0)		
Right upper limb	4 (40.0)	7 (70.0)	7 (70.0)		
Brunnstrom stage- upper limb ^b^; n (%)				6.000	0.199
I	5 (50.0)	2 (20.0)	5 (50.0)		
II	3 (30.0)	7 (70.0)	2 (20.0)		
III	2 (20.0)	1 (10.0)	3 (30.0)		
Brunnstrom stage- hand ^b^; n (%)				3.764	0.439
I	5 (50.0)	3 (30.0)	6 (60.0)		
II	3 (30.0)	6 (60.0)	2 (20.0)		
III	2 (20.0)	1 (10.0)	2 (20.0)		
Number of combined chronic disease ^b^; n (%)				1.505	0.826
0	1 (10.0)	2 (20.0)	2 (20.0)		
1	6 (60.0)	6 (60.0)	7 (70.0)		
2	3 (30.0)	2 (20.0)	1 (10.0)		
Main caregivers ^b^; n (%)				1.207	0.877
Nursing workers	5 (50.0)	7 (70.0)	7 (70.0)		
Offspring	3 (30.0)	2 (20.0)	2 (20.0)		
Spouse	2 (20.0)	1 (10.0)	1 (10.0)		

^a^ ANOVA was used for intergroup comparisons. ^b^ Chi-square test was used for intergroup comparisons

### Effects of the three interventions on elderly patients with stroke

All the outcome assessments at baseline and after intervention are presented in Table 2, and there was no significant difference between the three groups at baseline (*P* > 0.05). According to the intragroup comparisons, in all groups, self-esteem, daily life activity effectiveness, self-management effectiveness, total score of rehabilitation self-efficacy, positive emotion, upper limb function, and activities of daily living after intervention were significantly higher than those at baseline (Table 2). After intervention, the difference in the self-management effectiveness score was statistically significant among the three groups, and the results of Bonferroni indicated that the score of group A was significantly higher than that of Group B and Group C (*P* < 0.05). The difference in the rehabilitation self-efficacy score was statistically significant among the three groups after intervention. In accordance with the results of Bonferroni analysis, the score of group A was significantly higher than that of Group B and Group C (*P* < 0.05). Moreover, a significant difference in positive emotion among the three groups was observed, and the results of Bonferroni indicated that the score of group A was significantly higher than that of Group B and Group C (*P* < 0.05).

**Table 2:** The outcome assessments at baseline (T0) and after intervention (T1) (mean ± SD/median and quartile)

Outcomes	Time	Group A (*n* = 10)	Group B (*n* = 10)	Group C (*n* = 10)	*P* (intergroup)
Self-esteem	T0	22.30 ± 3.95	22.90 ± 2.96	22.40 ± 3.72	0.922^a^
	T1	27.50 ± 4.30	24.20 ± 2.39	24.10 ± 3.81	0.073 ^a^
*P* (intragroup)		0.006^c^	0.003^c^	0.000^c^	
Rehabilitation self-efficacy					
Daily life activity effectiveness	T0	10.40 ± 7.89	13.10 ± 6.23	9.60 ± 10.71	0.631 ^a^
	T1	20.80 ± 5.49	16.40 ± 3.98	15.90 ± 5.13	0.066 ^a^
*P* (intragroup)		0.103^c^	0.004^c^	0.003^c^	
Self-management effectiveness	T0	16.80 ± 8.26	13.80 ± 8.30	14.90 ± 7.49	0.702 ^a^
	T1	23.10 ± 5.82^#,†^	16.50 ± 5.72	17.20 ± 4.98	0.024 ^a^
*P* (intragroup)		0.004^c^	0.000^c^	0.000^c^	
Total score	T0	27.20 ± 13.96	26.90 ± 14.15	24.50 ± 16.25	0.905^a^
	T1	48.00 (35.75, 52.50)^#,†^	28.00 (24.75, 41.50)	30.00 (26.75, 43.25)	0.044^b^
*P* (intragroup)		0.005^d^	0.011^d^	0.033^d^	
Positive emotion	T0	17.00 (13.75, 23.00)	17.50 (12.75, 24.00)	14.00 (11.75, 22.50)	0.586 ^b^
	T1	32.80 ± 2.57^#,†^	24.30 ± 2.83	24.50 ± 2.84	< 0.000^a^
*P* (intragroup)		0.005^d^	0.005^d^	0.008^d^	
Upper limb function	T0	11.70 ± 5.25	13.30 ± 5.10	13.70 ± 7.60	0.742 ^a^
	T1	16.50 (15.00, 24.25)	13.50 (13.00, 18.50)	13.00 (11.75, 23.25)	0.082 ^b^
*P* (intragroup)		0.005^d^	0.007^d^	0.017^d^	
Activities of daily living	T0	37.00 (26.00, 42.75)	38.50 (31.25, 43.25)	30.00 (25.00, 46.75)	0.706 ^b^
	T1	48.30 ± 7.65	45.70 ± 5.21	43.10 ± 8.88	0.307 ^a^
*P* (intragroup)		0.008^d^	0.005^d^	0.005^d^	

^a^ ANOVA was used for intergroup comparisons. ^b^ Kruskal-Wallis H Test was used for intergroup comparisons. ^c^ A paired-sample t-test was used for intragroup comparisosns. ^d^ Wilcoxon signed rank sum test was used for intragroup comparisons. ^#^ Significant in comparison to B group: obtained from Bonferroni. ^†^ Significant in comparison to C group: obtained from Bonferroni

## Discussion

This study reports the results of a pilot study of a single-blinded, three-arm randomized controlled trial. A total of 36 stroke patients were recruited and 30 patients completed the trial. Through a three-week pilot trial, the levels of self-esteem, self-efficacy, positive emotion, activities of daily living and upper limb function of three groups showed significant increases. After intervention, the levels of self-management effectiveness, rehabilitation self-efficacy and positive emotion of Group A were significantly higher than those of Group C and Group B. In addition, recruitment, randomization, and assessment of patients were successful, meaning that the design was feasible and acceptable.

We found that all the outcome assessments of the three groups, including self-esteem, daily life activity effectiveness dimension and self-management effectiveness dimension of rehabilitation self-efficacy, total score of rehabilitation self-efficacy, positive emotion, upper limb function and activities of daily living, significantly improved after compared to the corresponding scores before the intervention. These findings suggested that usual rehabilitation treatment and care plus robot-assisted rehabilitation and reminiscent music therapy, usual rehabilitation treatment and care plus robot-assisted rehabilitation, and only usual rehabilitation treatment and care could all be effective for improving self-esteem, rehabilitation self-efficacy, positive emotion, upper limb function and activities of daily living in elderly patients after stroke. These are somewhat similar to other studies [41–43]. The reason may be that all three interventions could effectively improve upper limb function and activities of daily living in elderly stroke patients. When the motor function of stroke patients has been significantly improved and the rehabilitation effect is obvious, their psychological state may be improved accordingly [44, 45].

The results of intergroup comparison showed that the differences in the self-management effectiveness dimension of rehabilitation self-efficacy, rehabilitation self-efficacy total score, and positive emotion were statistically significant among the three groups after intervention, indicating that reminiscent music therapy assisted robot-assisted rehabilitation could better improve rehabilitation self-efficacy and positive emotions, which is somewhat similar to the results of previous studies [26, 46, 47]. The 100 reminiscent songs included in this study, which were released from 1935 to 1980, were selected through preliminary surveys to ensure that elderly stroke patients aged 60 and above were familiar with these songs when they were young, as memories from youth are more easily recalled [48]. Listening to familiar music can help stimulate conversations and memories about past experiences and events [27], enhance the recall of autobiographical memory and therapeutic reconstruction of the meaning of life [27], and improve the level of well-being in older people [49] Some scholars believe that recalling happy events helps to maintain a positive self to improve self-esteem and happiness, while recalling sad events helps to examine and explain past events to promote the ability to adapt to the existing environment [50, 51]. Therefore, patients are prompted to generate nostalgia through nostalgic music and think about past experiences, self-narratives, accomplishments, and problem solving, helping to improve self-efficacy and positive emotions [23]. In addition, a previous study showed that the combination of music therapy with family recollection can moderate physiological parameters and improve physiological stress responses [52], which may be beneficial for improving the psychological state of patients.

Although significant improvements were not observed in self-esteem, daily life activity effectiveness dimension, upper limb function and activities of daily living, trends of improvements were presented, which may be helpful for patients to participate in society and their functionality. Self-esteem is an individual’s positive evaluation and experience of self-worth, which is a core trait that affects psychological adaptation and development [53]. Changes or “loss” of self-esteem after stroke are common [54]. As a universal psychological activity, nostalgia has some positive functions of storing positive emotions and strengthening individual self-identity, and individuals can avoid environmental threats and restore positive self-esteem and self-efficacy through nostalgia [55]. A high level of self-esteem is associated with positive traits, including initiative, coping ability, and resilience when facing challenges, while individuals with low levels of self-esteem have more difficulty recovering from physiological illnesses [56]. The rehabilitation self-efficacy of stroke patients is a reflection of their rehabilitation ability and the self-confidence in self-management. Individuals with a high level of self-efficacy tend to adopt positive coping styles and actively solve problems to improve the disadvantaged situation [57]. Positive emotions and self-efficacy are considered as two important resources for individuals to actively cope with stress [58], which may help patients actively cope with rehabilitation treatment. In this study, reminiscent music therapy may evoke nostalgia in stroke patients, and encourage patients to look back on past experiences and their own feelings, thereby promoting joyful emotions and enhancing the value of life. Positive self-esteem, rehabilitation self-efficacy and positive emotion are beneficial for them to actively respond to their own status. These psychological traits are essential for patients to improve their subjective feelings of independent activities and reduce post-stroke fatigue, and actively respond to functional rehabilitation treatment, thus improving upper limb function and activities of daily living.

There are some limitations in this pilot study. The negative result may be related to the small sample size, which is a limitation to this study. A future study should recruit the required sample size calculated based on the results of this study to evaluate the intervention effect. West China Hospital of Sichuan University has shortened the hospitalization time of patients as much as possible, so the frequency of intervention was relatively low. Moreover, most patients live far away from the hospital and it is not convenient to return to the hospital after discharge, and scientific and accurate outcome measurements cannot be conducted. Therefore, this study did not consider the long-term effects of the intervention. It is necessary for future studies to clarity the long-term effects. Finally, group C was not time matched and had a lower intensity than the other two groups. The intervention protocol should be modified and the training intensity of group C can be changed to 1 h in the future study.

## Conclusions

This is the first pilot study to examine the benefits of reminiscent music therapy combined with robot-assisted rehabilitation on elderly patients with upper limb dysfunction after stroke. This pilot study is limited, in generalization, due to the small sample size. However, this pilot study has, for the first time, provided some preliminary empirical evidence of the practical significance of reminiscent music therapy combined with robot-assisted rehabilitation for significantly improving self-management effectiveness dimension, rehabilitation self-efficacy total score and positive emotion in elderly patients with upper limb dysfunction after stroke. Reminiscent music therapy combined with robot-assisted rehabilitation is a potentially promising approach, and rehabilitation environment with reminiscent music may help reduce patients’ negative emotions, which may prompt them to actively participate in rehabilitation training. Moreover, based on the results of the pilot study, the required sample size will be calculated, and a comprehensive randomized controlled trial will be conducted to scientifically evaluate the effectiveness of the intervention.

## Data Availability

The datasets generated and/or analysed during the current study are not publicly available due [REASON WHY DATA ARE NOT PUBLIC] but are available from the corresponding author on reasonable request.
